# Dietary Supplementation with Probiotics Alleviates Intestinal Injury in LPS-Challenged Piglets

**DOI:** 10.3390/ijms26157646

**Published:** 2025-08-07

**Authors:** Di Zhao, Junmei Zhang, Dan Yi, Tao Wu, Maoxin Dou, Lei Wang, Yongqing Hou

**Affiliations:** Hubei Key Laboratory of Animal Nutrition and Feed Science, Engineering Research Center of Feed Protein Resources of Agricultural By-Products, Ministry of Education, Wuhan Polytechnic University, Wuhan 430023, China; zhaodi24@163.com (D.Z.); zhangjunmei194012@163.com (J.Z.); yidan810204@163.com (D.Y.); wta005@163.com (T.W.); taoxian3515@163.com (M.D.); wanglei_wh@aliyun.com (L.W.)

**Keywords:** *Lactobacillales* spp., *Bacillus*, lipopolysaccharide, intestinal function

## Abstract

This study aimed to assess whether dietary supplementation with probiotics could alleviate intestinal injury in lipopolysaccharide (LPS)-challenged piglets. Healthy weaned piglets were randomly allocated to four individual groups (*n* = 6): (1) a control group; (2) an LPS group; (3) an LPS + *Lactobacillus* group; and (4) an LPS + *Bacillus* group. The control and LPS groups received a basal diet, while the probiotic groups were provided with the same basal diet supplemented with 6 × 10^6^ cfu/g of *Lactobacillus casei* (*L. casei*) or a combination of *Bacillus subtilis* (*B. subtilis*) and *Bacillus licheniformis* (*B. licheniformis*) at a dosage of 3 × 10^6^ cfu/g, respectively. On day 31 of the trial, overnight-fasted piglets were killed following the administration of either LPS or 0.9% NaCl solution. Blood samples and intestinal tissues were obtained for further analysis several hours later. The results indicate that dietary supplementation with probiotics significantly exhibited health-promoting effects compared with the control group and effectively reduced LPS-induced histomorphological damage to the small intestine, impairments in barrier function, and dysregulated immune responses via modulation of enzyme activity and the expression of relevant genes, such as *nuclear factor-kappa B* (*NF-κB*), *interleukin 4* (*IL-4*), *interleukin 6* (*IL-6*), *interleukin 10* (*IL-10*), *claudin-1*, nuclear-associatedantigenki-67 (*Ki-67)*, and *β-defensins-1* (*pBD-1*). Collectively, these results suggest that dietary supplementation with probiotics could alleviate LPS-induced intestinal injury by enhancing the immunity and anti-inflammatory responses in piglets. Our research provides a theoretical basis for the rational application of probiotics in the future.

## 1. Introduction

The intestine serves not only as the primary site for the digestion and absorption of nutrients but also plays a vital role as the initial defense barrier in systemic blood circulation, safeguarding the body against the invasion of both exogenous and endogenous harmful substances. It is widely acknowledged that weaned piglets are more vulnerable to environmental and physiological stresses due to their immature intestinal and immune systems, resulting in significant economic losses within the swine industry attributed to elevated morbidity and mortality rates.

For a long time, antibiotics have been employed as specific therapeutic agents to mitigate stress-induced injury and treat diseases. However, the emergence of serious adverse effects stemming from the overuse and misuse of antibiotics in the industry—such as drug resistance, residues, and environmental implications—has prompted a shift toward probiotics. Probiotics, defined as live microorganisms derived from animals or natural sources, have garnered considerable attention as a safe and effective alternative to in-feed antibiotics or as adjunctive treatments for intestinal diseases owing to their advantageous properties, including being residue-free, non-resistant, and non-toxic when incorporated into diets [1,2,3,4]. Probiotics have been recognized for their beneficial effects on gut health, immune function, and growth promotion in host organisms [5,6,7,8]. Numerous researchers have investigated the mechanisms of various probiotic strains to provide a theoretical foundation for further clinical applications both in vivo [5,9,10] and in vitro [11,12].

Four primary mechanisms have been identified regarding the action of probiotics [5,6,13]: (1) competing with the pathogenic bacteria for nutrients in the intestine; (2) competing with pathogens for binding sites in the intestinal environment; (3) producing toxins or antibacterial peptides; and (4) modulating the host’s immune response. Nonetheless, variations exist in the distribution of different strains within the intestines of animals as well as in the distribution and quantity of identical strains across different animal species. Therefore, the probiotics used in the present study were screened from several commercially available strains of *Bacillus subtilis*, *Bacillus licheniformis*, and *Lactobacillus* by comparing the characteristics of heat tolerance, acid tolerance, bile salt tolerance, and bacteriostasis. Additionally, preliminary experiments were conducted to determine the optimal ratio of *B. subtilis* to *B. licheniformis* through bacteriostatic testing.

In recent decades, *B. subtilis* and *B. licheniformis* have emerged as prominent subjects of probiotic research due to their capacity for anaerobic reproduction and their ability to germinate and proliferate within the intestinal environment. Studies have shown that dietary supplementation with *B. subtilis* and *B. licheniformis* could exert significant benefits on growth performance, nutrient digestion, caecal microbiota, and intestinal immunity in weaned piglets [14,15]. Additionally, *L. casei* strains have been shown to effectively maintain intestinal barrier function [12,13,16], mitigate intestinal inflammatory responses [4], and modulate gut microbiota and short-chain fatty acid production [17] in different models. Likewise, our previous study explicated that *L. casei* supplementation beneficially alleviated liver injury in an LPS-induced porcine model [18].

However, knowledge about the effects and mechanisms of the ‘*B. licheniformis* + *B. subtilis*’ complex and *L. casei* on intestinal function in weaning piglets under stressful conditions is still lacking. Animal models of LPS-induced inflammation were well-established in our previous study and shown to be a meaningful tool for studying immune stress in pigs [19]. Therefore, this study aimed to elucidate the impact of dietary supplementation with probiotics, specifically *L*. *casei* and a combination of *B*. *subtilis* and *B*. *licheniformis*, on intestinal injury in LPS-challenged piglets.

## 2. Results

### 2.1. Growth Performance and Diarrhea Incidence

The results on piglets’ growth performance, including average daily gain (ADG), feed/gain ratio (F:G), and diarrhea incidence, were published in our previous article, which reported the effects of probiotics on liver injury in LPS-challenged piglets [18]. Compared with the control group, dietary supplementation with *Bacillus* decreased (*p* < 0.05) the ratio of feed to gain (F:G). Moreover, dietary supplementation with *Lactobacillus* or *Bacillus* decreased (*p* < 0.05) the diarrhea incidence. However, there were no significant differences in the ADG between the control and probiotics groups.

### 2.2. Prostaglandin (PG) and Cortisol (COR) in Serum

**Figure 1:**
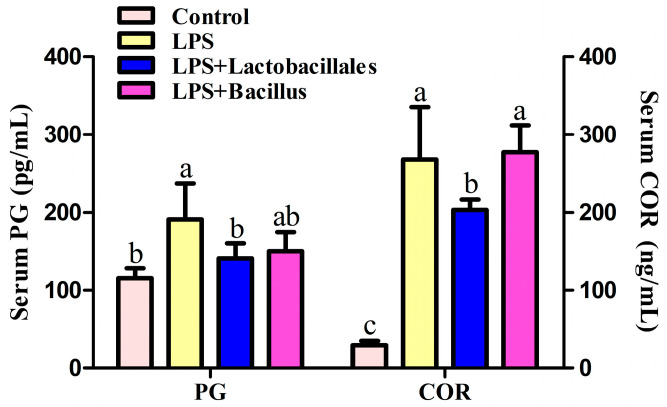
Concentrations of PG and COR in plasma. Data are presented as means ± SD, *n* = 6. Control group = piglets fed the basal diet and that received oral administration of saline; LPS group = piglets fed the basal diet and challenged with LPS; LPS + *Lactobacillus* group = piglets fed the basal diet supplemented with 6 × 10^6^ cfu/g *L. casei* and challenged with LPS; LPS + *Bacillus* group = piglets fed the basal diet supplemented with 3 × 10^6^ cfu/g *B. subtilis* and 3 × 10^6^ cfu/g *B. licheniformis* and challenged with LPS. Bars not sharing a common lowercase letter differ significantly (*p* < 0.05).

Data on the concentrations of PG and COR in each group are shown in Figure 1. Compared with the control group, LPS challenge resulted in a significant increase (*p* < 0.05) in the concentrations of PG and COR in plasma. However, dietary supplementation with *Lactobacillus* decreased (*p* < 0.05) the concentrations of PG and COR in plasma after LPS challenge and restored the PG concentration to the level of the control group.

### 2.3. D-Xylose Concentration and Diamine Oxidase (DAO) Activity in Plasma

**Figure 2:**
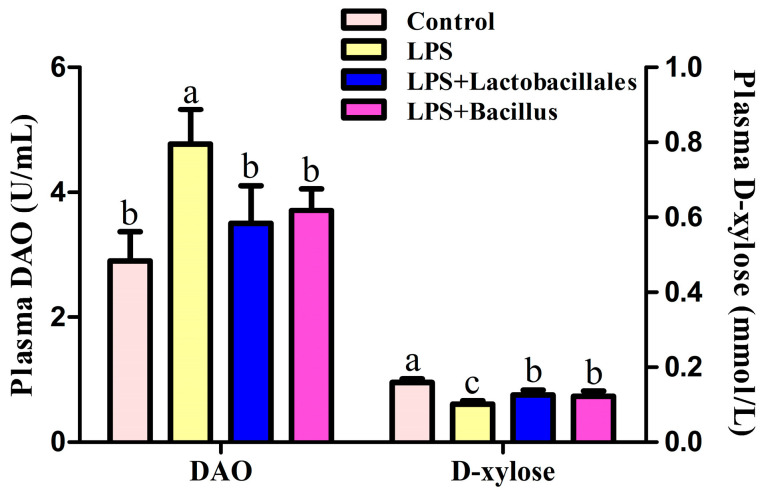
Concentration of D-xylose and activity of DAO in plasma. Data are presented as means ± SD, *n* = 6. Control group = piglets fed the basal diet and that received oral administration of saline; LPS group = piglets fed the basal diet and challenged with LPS; LPS + *Lactobacillus* group = piglets fed the basal diet supplemented with 6 × 10^6^ cfu/g *L. casei* and challenged with LPS; LPS + *Bacillus* group = piglets fed the basal diet supplemented with 3 × 10^6^ cfu/g *B. subtilis and* 3 × 10^6^ cfu/g *B. licheniformis* and challenged with LPS. Bars not sharing a common lowercase letter differ significantly (*p* < 0.05).

The concentration of D-xylose in the plasma of the LPS group decreased by 35.8%, while the activity of DAO increased by 64.4% compared with the control group, as shown in Figure 2. In comparison with the LPS group, dietary supplementation with *Lactobacillus* or *Bacillus* increased the concentrations of D-xylose (by 23.5% and 20.4%, respectively; *p <* 0.05) and decreased the activity of DAO (by 26.6% and 22.2%, respectively; *p <* 0.05) to the level of the control group.

### 2.4. Morphological Characterization of the Small Intestine

**Figure 3:**
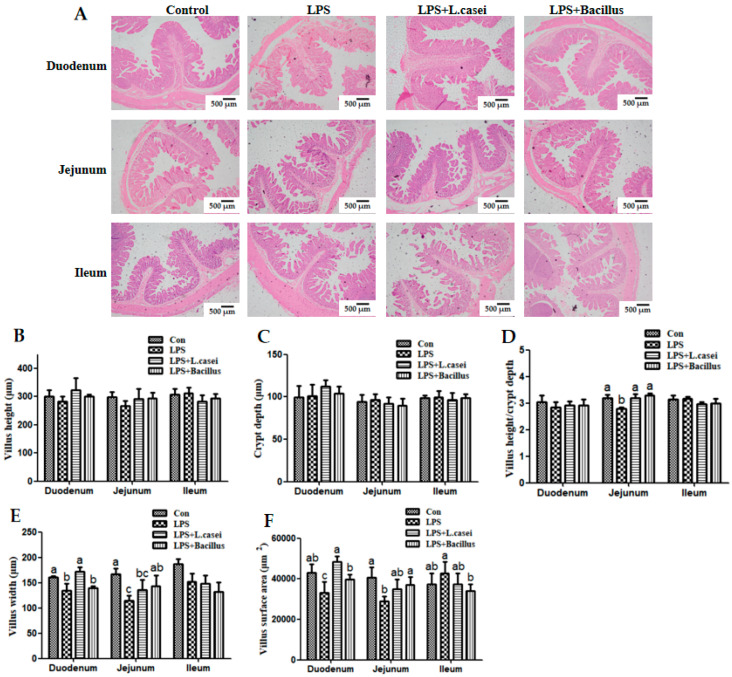
Morphological characterization of the small intestine (**A**–**F**). Scale bar: 500 μm. Data are presented as means ± SD, n = 6. 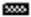 Control group = piglets fed the basal diet and that received oral administration of saline; 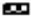 LPS group = piglets fed the basal diet and challenged with LPS; 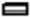 LPS + *Lactobacillus* group = piglets fed the basal diet supplemented with 6 × 10^6^ cfu/g *L. casei* and challenged with LPS; 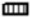 LPS + *Bacillus* group = piglets fed the basal diet supplemented with 3 × 10^6^ cfu/g *B. subtilis* and 3 × 10^6^ cfu/g *B. licheniformis* and challenged with LPS. Bars not sharing a common lowercase letter differ significantly (*p* < 0.05).

The effects of probiotics on the intestinal morphometry of LPS-challenged piglets are shown in Figure 3. The results show that LPS challenge caused a decrease (*p* < 0.05) in the ratio of villus height to crypt depth (VCR) of the jejunum (D), villus width of the duodenum and jejunum (E), and villus surface area of the duodenum and jejunum compared with the control group (F). Although LPS challenge decreased the villus height numerically in the duodenum and jejunum compared with the control group, the difference was not statistically significant due to the small number of animals per treatment group (B). In comparison with the LPS group, dietary supplementation with *L. casei* or *Bacillus* increased (*p* < 0.05) the ratio of villus height to crypt depth of the jejunum to the level of the control group (D). Dietary supplementation with *L. casei* also increased (*p* < 0.05) the villus width of the duodenum (E) and villus surface area of the duodenum to the level of the control group (F). Furthermore, dietary supplementation with *Bacillus* increased (*p* < 0.05) the villus width of the jejunum (E) as well as villus surface area of the duodenum and jejunum to the level of the control group (F).

### 2.5. Disaccharidase and Alkaline Phosphatase (ALP) Activity in the Small Intestine

**Figure 4:**
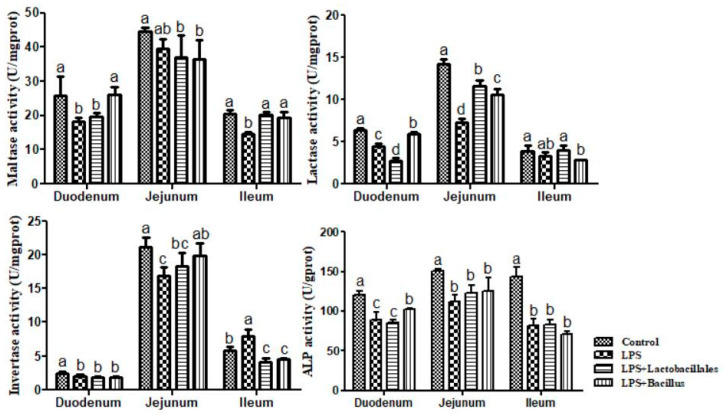
Disaccharidase and ALP activity in the small intestine. Data are presented as means ± SD, n = 6. 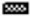 Control group = piglets fed the basal diet and that received oral administration of saline; 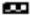 LPS group = piglets fed the basal diet and challenged with LPS; 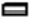 LPS + *Lactobacillus* group = piglets fed the basal diet supplemented with 6 × 10^6^ cfu/g *L. casei* and challenged with LPS; 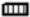 LPS + *Bacillus* group = piglets fed the basal diet supplemented with 3 × 10^6^ cfu/g *B. subtilis* and 3 × 10^6^ cfu/g *B. licheniformis* and challenged with LPS. Bars not sharing a common lowercase letter differ significantly (*p* < 0.05).

The results on disaccharidase and ALP activity in the small intestine are shown in Figure 4. LPS challenge resulted in a decrease (*p* < 0.05) in the activity of maltase in the duodenum and ileum. Compared with the LPS group, dietary supplementation with *Lactobacillus* and *Bacillus* increased (*p* < 0.05) the activity of maltase in the ileum to the level of the control group, and dietary *Bacillus* increased (*p* < 0.05) the activity of maltase in the duodenum. Results on lactase activity show that LPS challenge resulted in a decrease (*p* < 0.05) in the duodenum and jejunum. Compared with the LPS group, dietary supplementation with *Lactobacillus* increased (*p* < 0.05) the activity of lactase in the jejunum, and dietary supplementation with *Bacillus* increased (*p* < 0.05) the activity of lactase in the duodenum and jejunum. Additionally, LPS challenge resulted in a decrease (*p* < 0.05) in the activity of invertase in the duodenum and jejunum, while there was an increase (*p* < 0.05) in the ileum. Compared with the LPS group, dietary supplementation with *Lactobacillus* increased (*p* < 0.05) the activity of invertase in the ileum, and dietary supplementation with *Bacillus* increased (*p* < 0.05) the activity of invertase in the jejunum while decreasing (*p* < 0.05) the activity of invertase in the ileum.

Regarding intestinal ALP activity, LPS challenge resulted in a decrease (*p* < 0.05) in the activity of ALP in the small intestine. Compared to the LPS group, dietary supplementation with *Lactobacillus* had no significant effect in the small intestine as well as in the jejunum and ileum in the LPS + *Bacillus* group. However, dietary supplementation with *Bacillus* increased (*p* < 0.05) the activity of ALP in the duodenum.

### 2.6. mRNA Levels for Genes in the Small Intestine

**Figure 5:**
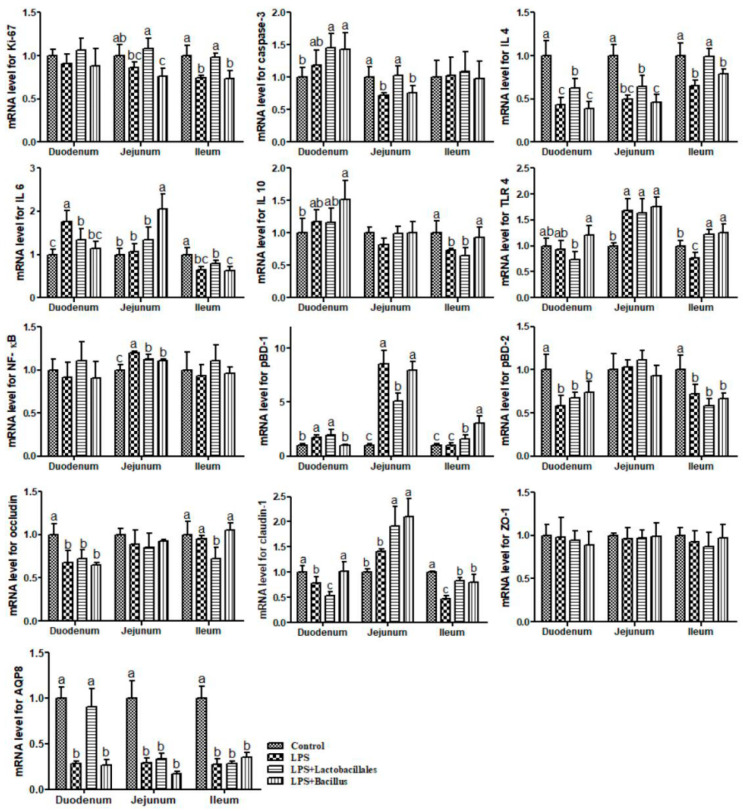
The mRNA levels for genes in the small intestine. Data are presented as means ± SD, n = 6. 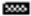 Control group = piglets fed the basal diet and that received oral administration of saline; 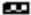 LPS group = piglets fed the basal diet and challenged with LPS; 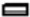 LPS + *Lactobacillus* group = piglets fed the basal diet supplemented with 6 × 10^6^ cfu/g *L. casei* and challenged with LPS; 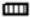 LPS + *Bacillus* group = piglets fed the basal diet supplemented with 3 × 10^6^ cfu/g *B. subtilis* and 3 × 10^6^ cfu/g *B. licheniformis* and challenged with LPS. Bars not sharing a common lowercase letter differ significantly (*p* < 0.05).

The results on mRNA levels for genes in the small intestine are shown in Figure 5. Compared with the control group, piglets in the LPS group exhibited significant increases (*p* < 0.05) in the mRNA levels of *IL-6* and *pBD-1* in the duodenum and *toll-like receptor 4* (*TLR4)*, *NF-κB*, and *pBD-1* in the jejunum. They also showed significant reductions (*p* < 0.05) in the mRNA levels of *IL-4*, *porcine β-defensin 2* (*pBD-2*), *occludin*, *claudin-1*, and *aquaporin 8* (AQP 8) in the duodenum, *caspase-3*, *IL-4*, and *AQP 8* in the jejunum, and *Ki-67*, *IL-4*, *IL-6*, *IL-10*, *TLR4*, *pBD-2*, *claudin-1*, and *AQP 8* in the ileum.

Piglets in the LPS + *Lactobacillus* group exhibited significant increases (*p* < 0.05) in the mRNA levels of *IL-4* and *AQP 8* in the duodenum, *Ki-67*, *caspase-3*, and *claudin-1* in the jejunum, and *Ki-67*, *IL-4*, *TLR4*, *pBD-1*, and *claudin-1* in the ileum. They also showed significant reductions (*p* < 0.05) in the mRNA levels of *IL-6* and *claudin-1* in the duodenum, *NF-κB* and *pBD-1* in the jejunum, and *occludin* in the ileum, when compared with the LPS group.

Additionally, compared with the LPS group, piglets in the LPS + *Bacillus* group exhibited significant increases (*p* < 0.05) in the mRNA levels of *claudin-1* in the duodenum, *IL-6* and *claudin-1* in the jejunum, and *IL10*, *TLR 4*, *pBD-1*, and *claudin-1* in the ileum. They also showed significant reductions (*p* < 0.05) in the mRNA levels of *IL-6* and *pBD-1* in the duodenum and *NF-κB* in the jejunum.

## 3. Discussion

Weaned piglets exhibit heightened susceptibility to increased rates of diarrhea and diminished growth performance due to their underdeveloped intestinal systems. However, our findings indicate that probiotic supplementation conferred beneficial effects, as evidenced by improved growth performance—reflected in a reduced feed-to-gain ratio in the Bacillus group—and a decrease in the incidence of diarrhea among weaned piglets, corroborating previous research [10,15,20].

The integrity of intestinal mucosal morphology and structure is essential for effective nutrient absorption and the maintenance of healthy growth in piglets. Our previous studies had demonstrated that LPS could induce intestinal morphology injury by inhibiting villus development [19]. As anticipated, similar intestinal injuries were observed following LPS administration, as indicated by reductions in the villus height-to-crypt depth ratio (VCR), villus width, and villus surface area across three intestinal segments. Notably, probiotics mitigated this injury by numerically restoring these indices to levels comparable to the control group, particularly in the duodenum and jejunum, aligning with previous findings [15,21]. These beneficial effects may largely stem from the ability of probiotics to inhibit LPS-induced autophagy in intestinal epithelial cells, as identified in the in vitro study by Han et al. (2016) [11]. This hypothesis is supported by the observed increases in mRNA levels of *caspase-3* and *Ki-67* in the small intestine of *L. casei*-treated subjects compared with the LPS group. In young pigs, both *caspase-3* and *Ki-67* are involved in maintaining enterocyte structure and function, facilitating the continuous replacement of enterocytes from crypt cells and producing protective mucins [22,23]. Our findings may provide new evidence for the role of the probiotic *L. casei* in the restoration of enterocyte injury induced by LPS, which may further enhance the integrity of intestinal mucosal morphology and structure in vivo.

Moreover, an increase in intestinal permeability is a significant indicator of compromised barrier function, which can predispose individuals to diarrhea and various other diseases. In the current study, LPS exposure was found to disrupt the integrity and permeability of the small intestine, resulting in elevated plasma levels of diamine oxidase (DAO), a reliable marker of intestinal mucosal permeability, and a reduction in D-xylose concentration, a sensitive indicator of intestinal integrity [19,22]. Intriguingly, the adverse effects induced by LPS were attenuated by probiotics, which led to increased D-xylose levels and decreased DAO activity. This finding underscores the efficacy of probiotics in preserving intestinal permeability. Furthermore, the observed increase in mRNA expression of *claudin-1* and *AQP 8* in probiotic-treated small intestinal mucosa further confirmed these beneficial effects. Consequently, it is plausible to assert that probiotics can ameliorate intestinal barrier dysfunction by upregulating *claudin-1* expression in LPS-treated piglets. These results align with previous studies that demonstrated the capacity of live probiotics to enhance epithelial barrier properties through the upregulation of *ZO-1* expression in intestinal epithelial cells [12,16].

Additionally, we investigated the impact of probiotics on nutrient absorption by evaluating the activity of digestive enzymes within the intestinal mucosa. Maltase, sucrase, and lactase are three critical disaccharidases essential for carbohydrate digestion and absorption. Deng et al. (2017) [24] reported an increase in maltase and sucrase activity in rats treated with probiotics, particularly those receiving *L. bacillus*. Consistently, our observations revealed enhanced activities of these enzymes in probiotic-treated piglets, which may also elucidate the improvements in intestinal histomorphology and barrier function noted earlier. Moreover, we identified a positive regulatory effect of probiotics, especially the bacillus complex, in alleviating the adverse effects induced by LPS through the enhancement of ALP activity in the jejunum. Hu et al. (2015) [25] indicated that elevated ALP levels confer beneficial effects on intestinal cell proliferation and absorption. Therefore, our findings suggest that dietary supplementation with probiotics may mitigate the severity of nutrient digestion and absorption impairments caused by LPS, potentially leading to improved growth performance in pigs. It is hypothesized that this effect may arise from the modulation of intestinal pH by probiotics, thereby enhancing enzyme activity. However, this hypothesis necessitates further in-depth research to fully elucidate the underlying mechanisms.

The proposed mechanism by which probiotics confer protection on the small intestine in LPS-challenged piglets may be linked to modifications in genes associated with the intestinal mucosal immune response. It is well-established that LPS challenges can elicit a pronounced inflammatory response within the intestine, primarily mediated through the TLR 4–NF-κB signaling pathway, which promotes the synthesis of pro-inflammatory mediators and cytokines, including *COR*, *TNF-α*, and *IL-6* [26,27]. Our observations revealed a significant upregulation of *TLR 4* and *NF-κB* gene expression in the jejunal mucosa, indicating the activation of the *TLR4*–*NF-κB* signaling pathway following LPS exposure. Furthermore, levels of the pro-inflammatory cytokine *IL-6*, PG, and COR were found to be elevated in the duodenum and bloodstream, respectively [28,29,30], while the anti-inflammatory cytokines *IL-4* and *IL-10* exhibited a reduction in various segments of the small intestine. These findings confirm the successful establishment of our inflammation model.

Notably, pre-administration of probiotics appeared to exert beneficial effects on the inflammatory response in stressed piglets by enhancing the levels of the anti-inflammatory cytokines *IL-4* and *IL-10* while concurrently reducing *IL-6* in the duodenum, *NF-κB* in the jejunum, and PG and COR in the blood, thereby suggesting a mitigation of inflammatory injury. Interestingly, probiotics did not influence the expression of the *TLR4* gene under the conditions of our experiment, which may be because probiotics affect the translational regulation of the *TLR4* gene. Additionally, probiotics were found to enhance innate immunity by downregulating the expression of *pBD-1* in both the duodenum and jejunum compared with the LPS group [31]. Consistent with our findings, several prior studies have documented the beneficial roles of probiotics in reducing inflammation and exerting immunostimulatory effects in both cellular models [32] and piglets [33]. Mazziotta et al. (2023) [6] reported that *L. casei* contributes to health-promoting and immunomodulatory properties by increasing levels of anti-inflammatory cytokines such as IL-10. Moreover, probiotic complexes have been shown to alleviate inflammation by downregulating the gene expression of *IL-6* and *pBD-2* in LPS-challenged rats [24] and piglets [34], respectively. However, it is noteworthy that the expression of the *pBD-2* gene was unexpectedly downregulated following LPS challenge. We hypothesize that this unanticipated change in *pBD-2* expression may be attributed to the abrupt alterations in intestinal microbiota induced by LPS [6] as well as the differential distribution patterns of the two defensins [35]. Consequently, further validated experiments are warranted to comprehensively elucidate the underlying mechanisms at the protein level in future studies.

In conclusion, the application of probiotics (6 × 10^3^ cfu/g *L. casei* or a 6 × 10^3^ cfu/g combination of *B. licheniformis* and *B. subtilis*) prior to treatment improved growth performance, as evidenced by a reduced F/G and diarrhea rate. Additionally, these probiotics positively influenced the digestive and absorptive functions of nutrients, as indicated by improvements in intestinal mucosal morphology and structure, elevated activities of intestinal disaccharidases and ALP, an increase in the mRNA levels of *AQP 8*, and higher plasma concentrations of D-xylose. Furthermore, the probiotics contributed to the integrity of the intestinal barrier, as demonstrated by decreased plasma concentrations of DAO and increased intestinal mRNA levels of *claudin-1* and *Ki-67*. The immune response was also enhanced, as reflected by elevated mRNA levels of *IL-4* and *IL-10*, reduced mRNA levels of intestinal *IL-6*, *NF-κB*, and *pBD-1*, and decreased blood concentrations of PG and COR in pigs subjected to LPS challenge. The multifaceted effects of the probiotics were primarily mediated through the *TLR4*–*NF-κB* signaling pathway.

## 4. Materials and Methods

### 4.1. Experimental Animals and Design

The research protocol for this study was approved by the Institutional Animal Care and Use Committee at Wuhan Polytechnic University (WPU202404004, June 2024). Thirty-two crossbred healthy pigs (Durc × Landrace × Yorkshire) were reared by sows and weaned at 21 days of age (day 0 of the trial). On day 1 of the trial, after a 3-day adaptation period, piglets (25 days of age, average body weight of 5.03 ± 0.59 kg) were randomly assigned to one of four groups (*n* = 6): (1) a control group; (2) an LPS group; (3) an LPS + *Lactobacillales* group; (4) an LPS + *Bacillus* group. Each piglet was individually caged in a pen with dimensions of 1.20 × 1.10 m in a temperature-controlled nursery barn (22 to 25 °C). Free access to feed and water was provided.

During days 1–30 of the trial (25–54 days of age), piglets in the control and LPS groups were fed a basal diet, while the two probiotic groups were fed the basal diet supplemented with 6 × 10^6^ cfu/g *L. casei* or a *bacillus* complex consisting of 3 × 10^6^ cfu/g *B. subtilis* and 3 × 10^6^ cfu/g *B. licheniformis*. The dose of probiotics used for the present study was based on previous work indicating that 6 × 10^6^ cfu/g *L. casei* and a mixture of 3 × 10^6^ cfu/g *B. subtilis* and 3 × 10^6^ cfu/g *B. licheniformis* have effective antibacterial activity in vitro and health-promoting effects on piglets [18].

The basal diet (Table 1) was formulated to meet National Research Council (NRC) requirements for all nutrients [36]. *L. casei* and the *bacillus* complex (powder) were mixed thoroughly with the basal diet in a one-batch mix. *L. casei* was grown overnight at 37 °C in MRS broth (Cat. HB0384-1, Oxoid, Haibo, Qingdao, China), while *B. subtilis* and *B. licheniformis* were grown overnight at 37 °C in LB broth (Cat. HB0128, Oxoid, Haibo, China). After overnight culture, probiotics were centrifuged at 3000 rpm for 10 min at room temperature. The powders of *L. casei, B. subtilis*, and *B. licheniformis* were obtained by vacuum-drying and then mixed with feed according to their respective concentration requirements.

On day 31 of the trial (55 days of age), all piglets in the four groups were fasted overnight. Then, piglets from the LPS, LPS + *Lactobacillus*, and LPS + *Bacillus* groups were intraperitoneally administered LPS (*E. coli* serotype 055: B5; Sigma Chemical Inc., St. Louis, MO, USA) at a dose of 100 μg/kg BW [19]. The control group received the same volume of 0.9% NaCl solution. Two hours later, all piglets were orally administered D-xylose at a dose of 0.1 g/kg BW [22,27]. Three hours after the LPS or saline injection (1 h after D-xylose infusion), blood samples were collected from the anterior vena cava into heparinized vacuum tubes and centrifuged at 3500× *g* for 10 min at 4 °C to obtain plasma [37], which was then stored at −80 °C until analysis. Six hours later, all pigs were anesthetized with intravenous injection of sodium pentobarbital (50 mg/kg BW) to obtain intestinal tissues. The pig’s abdomen was immediately opened from the sternum to the pubis, and the entire gastrointestinal tract was exposed directly [22,27]. The small intestine was dissected free of the mesentery and placed on a chilled stainless steel tray. Two intestinal segments (one 5 cm piece for intestinal histological measurements and one 15 cm piece for intestinal tissue collection) were cut at the distal duodenum, mid-jejunum, and mid-ileum, respectively. The 5 cm sections were gently flushed with ice-cold phosphate-buffered saline (PBS, pH 7.4) and placed in fresh 4% paraformaldehyde/phosphate-buffered saline for further intestinal histological measurements [27]. The 15 cm sections were flushed with ice-cold PBS (pH 7.4) to remove intestinal contents and then cut into pieces with scissors after drying with filter paper. The procedure was conducted with a sterile glass microscope slide at 4 °C. Then, samples were subpackaged into 6 small portions, rapidly placed in liquid nitrogen to freeze them, and transferred to a freezer at −80 °C for future analyses. All samples were collected within 10 min after killing. Throughout the trial, growth indices, including body weight, feed intake, and diarrhea incidence, of piglets were observed and statistically analyzed to evaluate their growth performance.

### 4.2. Prostaglandin (PG) and Cortisol (COR) in Serum

PG and COR concentrations in the serum were determined using a commercially available ^125^I RIA kit (Beijing North Institute of Biological Technology, Beijing, China) that had been previously validated for use in pigs [27]. The detection limit for the PGE2 assay was 0.12 pg/mL, while the detection limit for the COR assay was 0.2 ng/mL The intra-assay and inter-assay coefficients of variation for PGE2 were less than 7.5% and less than 10.5%, respectively. The intra-assay and inter-assay coefficients of variation for COR were less than 10% and less than 15%, respectively.

### 4.3. D-Xylose Concentration and Diamine Oxidase (DAO) Activity in Plasma

The concentration of D-xylose and DAO activity in plasma were determined using spectrophotometry with a commercially available kit (Nanjing Jiancheng Bioengineering Institute, Nanjing, China). The measurements are expressed as μg/mL and U/mL, respectively. Briefly, to measure D-xylose activity, plasma samples were added to a phloroglucinol color regent solution and heated for 4 min at 100 °C. A D-xylose standard solution was prepared by dissolving D-xylose in saturated benzoic acid to obtain concentrations of 0, 0.7, 1.3, and 2.6 mM. The D-xylose standard solutions or plasma samples were then added to the color reagent solution, and then the absorbance at 554 nm was determined using a spectrophotometer (Model 6100, Jenway Ltd., Dumfries, UK). The standard solution of 0 mmol/L D-xylose was considered as the blank. To measure DAO activity, an assay mixture (3.8 mL) containing 3 mL of phosphate buffer (0.2 M, pH 7.2), 0.1 mL (0.004%) of horseradish peroxidase solution (Sigma Chemicals, Shanghai, China), 0.1 mL of o-dianisidine-methanol solution (0.5% of o-dianisidine in methanol), 0.5 mL of plasma or intestinal homogenate, and 0.1 mL of substrate solution (0.175% of cadaverine dihydrochloride, Sigma Chemicals, Shanghai, China) was prepared. The mixture was then incubated for 30 min at 37 °C, and the absorbance at 436 nm was measured to indicate DAO activity [22].

### 4.4. Intestinal Morphology Analysis

To determine intestinal morphology, three paraformaldehyde-fixed intestinal segments (from the duodenum, jejunum, and ileum) were dehydrated and embedded in paraffin. Five-micrometer segments were cut and then stained with hematoxylin and eosin stain. Intestinal morphology was determined using a light microscope (Olympus BX43, Tokyo, Japan) with the Olympus cellSens image analysis software (cellSens Standard software, version 1.18). Villus height (the distance from the villus tip to the crypt mouth) and width (the distance of the widest villi), crypt depth (the distance from the crypt mouth to the base), and villous surface area were measured. The ratio of villus height to crypt depth was calculated later. All intestinal histological analysis was done by the same person, who was blinded to the treatments.

### 4.5. Disaccharidase Activity in the Small Intestine

Disaccharidase activity was determined by using commercially available kits (Cat. A082-1, A082-2, and A082-3 for lactase, sucrase, and maltase, respectively; Nanjing Jiancheng Bioengineering Institute, Nanjing, China) via spectrophotometry, as previously described [38]. Briefly, 10 µL of double-distilled water, glucose standard solution (5.55 mmol/L), or test sample (supernatant fluid collected from the homogenate with PBS) was added to a test tube and co-incubated for 20 min at 37 °C with 20 µL of the respective substrate. Thereafter, 10 µL of terminating agent and 1 mL of chromogenic agent were added, followed by 15 min of incubation at 37 °C. Double-distilled water was used to set zero optical density at 505 nm, and optical density values for each tube were then measured. One unit (U) of enzyme activity was defined as 1 nmol substrate hydrolyzed per minute under assay conditions (37 °C, pH 6).

### 4.6. Activity of Intestinal Mucosa Alkaline Phosphatase (ALP)

ALP activity was determined using commercially available kits (Cat. A059-1, Nanjing Jiancheng Bioengineering Institute, Nanjing, China) via spectrophotometry, as previously described by [39]. Briefly, 30 µL of double-distilled water, phenol standard solution (0.1 mg/mL), or test sample (supernatant fluid collected from the homogenate with PBS) was added to a test tube and incubated with 0.5 mL of buffer and 0.5 mL of the respective substrate for 15 min at 37 °C. Afterward, 1.5 mL of chromogenic agent was added and immediately mixed to measure the optical density. The optical density of each tube was then read, setting zero optical density at 520 nm with double-distilled water. One unit (U) of enzyme activity was defined as 1 nmol substrate hydrolyzed per minute under assay conditions (37 °C, pH 6).

### 4.7. Quantitative Real-Time PCR Analysis of mRNA

The expression of genes in the intestine was quantified using quantitative Real-Time PCR (qRT-PCR). A frozen tissue sample (~100 mg) of the small intestine was powdered under liquid nitrogen using a mortar and pestle. The RNAiso Plus kit (Cat. 9108/9109, TaKaRa, Dalian, China) was used for total RNA extraction and the extracted RNA was then dissolved in RNase-free water. The integrity of the RNA was confirmed by agarose gel electrophoresis. The extracted RNA was used for RT-PCR analysis only if the 28 S/18 S rRNA ≥ 1.8. The purity and concentration of the RNA were determined using a NanoDrop^®^ 2000 spectrophotometer (Thermo Scientifc, Wilmington, DE, USA) based on the OD260/OD280 ratio. All ratios of OD260/OD280 for samples were above 1.8, indicating that the purity of nucleic acids was equal to 90–100% [27,40]. The RNA was reverse-transcribed into cDNA using a PrimeScript^®^ RT reagent kit with gDNA eraser (Cat. RR047A, Takara, Dalian, China), and the synthesized cDNA was stored at −20 °C until use. The expression of the target genes was analyzed by qRT-PCR with an Applied Biosystems 7500 Real-Time PCR System (Applied Biosystems, Life Technologies, Foster City, CA, USA) with SYBR^®^ Premix Ex Taq^TM^ (Tli RNaseH Plus) qPCR kit (Cat. RR420A, TaKaRa, Dalian, China), following the manufacturer’s instructions. The primer sequences and accession numbers are listed in Table 2. In brief, the reaction mixture contained 10.0 µL of SYBR Premix ExTaq, 0.4 µL of ROX reference dye II (50×), 2.0 µL of cDNA, 0.4 µL of forward primer (10 µmol/L), 0.4 µL of reverse primer (10 µmol/L), and 6.8 µL of RNase-free water in the 20 µL PCR reaction. The PCR program was run as follows: 95 °C for 30 s, followed by 40 cycles of 95 °C for 5 s and 60 °C for 31 s. A subsequent melting curve analysis (95 °C for 15 s, 60 °C for 1 min, and 95 °C for 15 s) with continuous fluorescence measurement and final cooling to room temperature was performed to validate primer specificity. The mRNA abundance of the target genes was normalized to the housekeeping gene (porcine ribosomal protein L4, RPL4). The relative mRNA expression of genes was calculated as the ratio of the target gene to the control gene using the 2^−∆∆Ct^ method. Each biological sample was run in triplicate.

### 4.8. Statistical Analysis

Experimental data, expressed as means ± SD, were analyzed using one-way analysis of variance. The normality and constant variance of the data were tested using Levene’s test [41]. Differences among treatments were evaluated using Duncan’s multiple comparison test. All statistical analyses were performed using SPSS 20.0 software (SPSS Inc., Chicago, IL, USA). Probability values less than 0.05 were considered statistically significant.

## Figures and Tables

**Table 1:** Composition and nutrient contents of the basal diet (air-dry basis).

Items	Content	Items	Content
Ingredient (%)		Nutrient composition	
Corn	61.88	Digestion energy ^†^ (MJ/kg)	14.22
Soybean meal	21.98	Crude protein ^§^ (%)	20.9
Wheat middlings	4.00	Total threonine ^†^ (%)	0.74
Whey powder	3.00	Total methionine ^†^ (%)	0.30
Fish meal	3.00	Total methionine + cystine ^†^ (%)	0.65
Soya protein concentrate	1.50	Total lysine ^†^ (%)	1.15
CaHPO_4_	1.25	Total tryptophan ^†^ (%)	0.21
Premix *	1.00	Ca ^§^ (%)	0.70
Limestone	0.69	Total P ^§^ (%)	0.60
Acidifier	0.30	Available P ^†^ (%)	0.32
NaCl	0.30		
Mold inhibitor	0.10		
Soybean oil	0.50		
Choline chloride	0.20		
L-Lysine·HCl (78.8% lysine)	0.25		
_DL_-Methionine (99% methionine)	0.05		
Total	100.00		

* The premix provides the following amounts of trace minerals and vitamins per kg of the complete diet: Fe, 100 mg (FeSO_4_.H_2_O); Cu, 150 mg (CuSO_4_.5H_2_O); Zn, 100 mg ZnSO_4_.7H_2_O; I, 0.5 mg (KI); Se, 0.3 mg (Na_2_SeO_3_.5H_2_O); vitamin A, 1,0800 IU (3.66 mg); vitamin D_3_, 4000 IU (0.10 mg); vitamin E, 40 IU (36.4 mg); vitamin K_3_, 4 mg; vitamin B_1_, 6 mg; vitamin B_2_, 12 mg; vitamin B_6_, 6 mg; vitamin B_12_, 0.05 mg; biotin, 0.2 mg; folic acid, 2 mg; niacin, 50 mg; _D_-calcium pantothenate, 25 mg. ^†^ Calculated value. ^§^ Analyzed value.

**Table 2:** Primers sequences used for quantitative Real-Time PCR analysis.

Genes	Forward (5′-3′)	Reverse (5′-3′)	Annealing Temperatures (°C)	Accession Numbers	Primer’s Efficiency (%)
*Ki-67*	CGCAACCAAGCAAC	ACAGTGCCAAACTGGGAGAAA	60	NM_001101827	106
*Caspase-3*	GAACTCTAACTGGCAAACCCAAA	GTCCCACTGTCCGTCTCAATC	60	NM_214131.1	92
*IL-4*	TACCAGCAACTTCGTCCAC	ATCGTCTTTAGCCTTTCCAA	60	NM_214123.1	89
*IL 6*	TACTGGCAGAAAACAACCTG	GTACTAATCTGCACAGCCTC	60	NM_214399.1	93
*IL 10*	CGGCGCTGTCATCAATTTCTG	CCCCTCTCTTGGAGCTTGCTA	60	NM_214041.1	103
*TLR 4*	GCCTTTCTCTCCTGCCTGAG	AGCTCCATGCATTGGTAACTAATG	60	NM_001113039.2	94
*NF-κB*	CTCGCACAAGGAGACATGAA	ACTCAGCCGGAAGGCATTAT	60	NM_001048232.1	107
*pBD-1*	ACCGCCTCCTCCTTGTATTC	CACAGGTGCCGATCTGTTTC	60	NM_213838	94
*pBD-2*	TTGCTGCTGCTGACTGTCTG	CTTGGCCTTGCCACTGTAAC	60	NM_214442	91
*Occludin*	TATGAGACAGACTACACAACTGGCGGCGAGTCC	ATCATAGTCTCCAACCATCTTCTTGATGTG	60	XM_005672522.3	93
*Claudin-1*	GGTGCCCTACTTTGCTGCTC	CCCACACGGTTTTGTCCTTT	60	NM_001244539.1	108
*ZO-1*	AGGCGATGTTGTATTGAAGATAAATG	TTTTTGCATCCGTCAATGACA	60	CK453343	92
*AQP8*	TGTGTCTGGAGCCTGCATGAAT	AGCAGGAATCCCACCATCTCA	60	NM_001112683.1	108
*RPL4*	GAGAAACCGTCGCCGAAT	GCCCACCAGGAGCAAGTT	60	XM_005659862.3	102

## Data Availability

The original contributions presented in this study are included in the article. Further inquiries can be directed to the corresponding author.
